# Risk Assessment of Mercury-Contaminated Fish Consumption in the Brazilian Amazon: An Ecological Study

**DOI:** 10.3390/toxics11090800

**Published:** 2023-09-21

**Authors:** Paulo Cesar Basta, Ana Claudia Santiago de Vasconcellos, Gustavo Hallwass, Decio Yokota, Daniel de Oliveira d’El Rei Pinto, Danicley Saraiva de Aguiar, Ciro Campos de Souza, Marcelo Oliveira-da-Costa

**Affiliations:** 1 Department of Endemic Diseases Samuel Pessoa, National School of Public Health Sergio Arouca, Oswaldo Cruz Foundation, Rio de Janeiro 21041-210, RJ, Brazil; daniel@habitatgeo.com.br; 2 Laboratory of Professional Education on Health Surveillance, Joaquim Venâncio Polytechnic School of Health, Oswaldo Cruz Foundation, Rio de Janeiro 21040-900, RJ, Brazil; ana.vasconcellos@fiocruz.br; 3 Institute of Science, Technology and Innovation, Graduate Studies in Applied Ecology, Federal University of Lavras, São Sebastião do Paraíso 37950-000, MG, Brazil; gustavo.hallwass@ufla.br; 4 Iepé—Institute for Indigenous Research and Education, Macapá 68908-120, AP, Brazil; decio@institutoiepe.org.br; 5 Greenpeace Brasil, São Paulo 05509-006, SP, Brazil; danicley.aguiar@greenpeace.org; 6 ISA—Socioambiental Institute, São Paulo 01047-912, SP, Brazil; ciro@socioambiental.org; 7 WWF-Brasil, Brasília 70377-540, DF, Brazil; marcelo@wwf.org.br

**Keywords:** Amazon, fish, health risk assessment, mercury, mining

## Abstract

Mercury is one of the most dangerous contaminants on the planet. In recent years, evidence of mercury contamination in the Amazon has significantly increased, notably due to gold-mining activities. Although mercury contamination in fish has consistently been documented, little is known about the risk associated with fish consumption by populations in urban areas of the Amazon. We sampled 1010 fish sold in public markets in six state capitals and 11 additional cities. Mercury levels were determined for each specimen, and the evaluation of the health risks associated with consuming mercury-contaminated fish was conducted according to the methodology proposed by the World Health Organization (WHO). Our study reveals that more than one-fifth (21.3%) of the fish sold in urban centers had mercury levels above the safe limits (≥0.5 µg/g) established by the Brazilian Health Surveillance Agency (ANVISA). The prevalence of Hg contamination ≥0.5 µg/g was approximately 14 times higher in carnivorous than in noncarnivorous fish. The analysis of the risk attributable to fish consumption reveals that daily mercury intake exceeded the reference dose recommended by the U.S. EPA in all population groups analyzed, reaching up to 7 and 31 times in women of childbearing age and children from 2 to 4 years old, respectively. However, these risks are diverse depending on the type of fish consumed and must be considered to formulate appropriate nutritional guidelines for safe fish consumption by the local community.

## 1. Introduction

Mercury ranks third in the world for toxicity among the environmental pollutants that are most dangerous to human health. Approximately 19 million people around the world have been estimated to be at risk of becoming sick due to contact with this chemical contaminant. Artisanal gold mining is the largest source of human exposure to mercury in Latin America [1,2]. In response, the United Nations promulgated the Minamata Convention in 2013, which aimed to ban mercury from all industrial processes on the planet and regulate informal mining to control and replace the use of mercury. In Brazil, the Minamata Convention was promulgated by Decree 9470 on 14 August 2018. However, efforts to contain this threat since then have not been sufficient to control the gold-mining boom in the Brazilian Amazon, especially in the last five years.

Over time, metallic mercury used in gold mining accumulates in river sediments, where it is converted into methylmercury (the most dangerous chemical form to human health and the ecosystem) and is quickly incorporated into the organisms that make up the aquatic biota [3,4,5,6]. Much of the danger associated with methylmercury is due to its high neurotoxic potential and its ability to bioaccumulate and biomagnify in aquatic food chains. Fish are directly affected, resulting in serious health damage to humans and various animals that consume these and other contaminated aquatic organisms.

Methylmercury is highly liposoluble, and due to this characteristic, it can cross the blood–brain barrier and reach the central nervous system. The main health damages caused by methylmercury are the following: changes in gait, problems with balance and motor coordination, decreased visual field, and loss of skin sensitivity [7,8]. In pregnant women, contamination is even more serious since methylmercury is capable of crossing the placental barrier and reaching the developing fetus’s brain, causing irreversible damage, including hearing loss, cognitive deficits, developmental delays, and congenital malformations in children exposed during the intrauterine period [9,10,11].

The Amazonian populations have one of the highest rates of per capita fish consumption in the world [12,13,14]. Fish is the animal protein that is most easily accessible in the Amazon, ensuring the food and nutritional security of riverine and urban populations in the region. Fish is a food with high nutritional value due to its high protein content and its inclusion of important vitamins and minerals for maintaining good health [15,16]. Despite numerous pieces of evidence regarding the nutritional quality of fish, the increasing contamination of aquatic systems with environmental pollutants such as pesticides and heavy metals has raised concerns in society and sparked an important debate about the risks and benefits of a diet rich in this type of animal protein.

The contamination of fish in the Amazon Basin via gold mining and other sources has been well documented since the 1960s. In recent years, evidence of increased mercury contamination in fish in the rivers that form the Amazon basin has significantly increased due to the growth of mining activity. This has raised a series of concerns about the health of the population living in the region [6,17,18].

Considering the increase in gold-mining activity in recent years, as well as the severity of health damage that mercury can cause to both the population and the environment, this study evaluated the risk associated with fish consumption by populations in urban areas of the Brazilian Amazon. Based on the findings of this study, we hope to broaden the debate about the deleterious effects caused by gold mining not only for riverside and traditional populations, but also for populations living in urban centers who also have the cultural habit of consuming large amounts of fish from the region.

## 2. Materials and Methods

### 2.1. Area and Study Design

**Figure 1:**
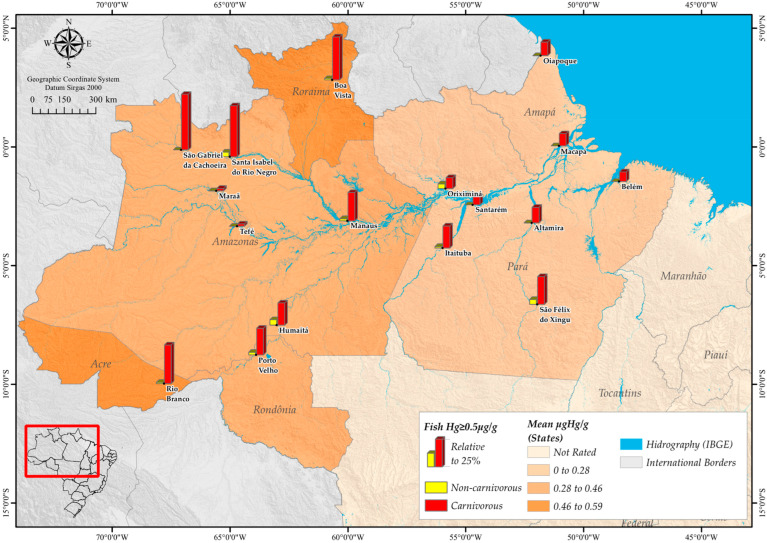
Spatial distribution of average levels of mercury contamination in analyzed fish (average between carnivorous and noncarnivorous fish) by Federative Unit and according to prevalence of contamination ≥0.5 µg/g considering the 17 collection points, Amazon Basin, Brazil, 2021–2022.

An ecological approach was undertaken to assess the health risks associated with fish consumption in six states of the Brazilian Amazon, including state capitals and 11 additional cities, totaling 17 municipalities: Rio Branco, Macapá, Oiapoque, Humaitá, Manaus, Maraã, Santa Isabel do Rio Negro, São Gabriel da Cachoeira, Tefé, Altamira, Belém, Itaituba, Oriximiná, Santarém, São Félix do Xingú, Porto Velho, and Boa Vista (Figure 1).

### 2.2. Fish Sampling and Mercury Analysis

The sampled fish were acquired from public markets, open-air markets, or directly from fishermen at the fishing landing points between March 2021 and September 2022. To standardize the sampling, a preliminary list was prepared considering the fish commonly found in open-air markets and markets in the regions [19,20,21,22], their priority feeding habits, and their trophic guild [22,23,24]. We prioritized at least three different species in each trophic guild and at least three individuals of different sizes for each species. The trophic guilds were generally classified as carnivorous, omnivorous, detritivorous, and herbivorous.

After being acquired, the fish were placed in thermal boxes with ice and sent to the characterization/description stage, after which muscle tissue samples were obtained to determine mercury levels. Each of the specimens was photographed, with the following information recorded: common name, scientific name, date, location of fish capture, location of fish purchase, weight (g), and standard length (cm). Each collected fish had its identification confirmed to the lowest possible taxonomic level using specialized literature, dichotomous keys, and consultation with experts.

Subsequently, approximately 20 g of muscle tissue was extracted from the dorsal part of each fish specimen, which was stored in Ziploc plastic bags and properly identified with a code representing the name of the species, location of acquisition, and date of collection. The samples were sent to the Laboratory of Environmental Mercury Speciation of the Mineral Technology Center (LEMA/CETEM) and to the Laboratory of Mercury of The Evandro Chagas Institute, Surveillance Health and Environment Secretariat of the Ministry of Health for total mercury analysis. At the laboratories, aliquots of 3.0 g of wet muscle tissue were precisely weighed, and three replicates were performed per fish sample. The analysis accuracy with the replicates was above 85% (calibration curve R: 0.9949), and the recovery with the reference material IAEA-476 (0.578 mg/kg) was 94%. The analytical technique employed was atomic absorption spectrometry with a graphite furnace (equipment: RA-915+ coupled with Py-ro-915+—Serial number 465). The detection limit (DL) for mercury was 0.0005 mg/kg, and the quantification limit (QL) was 0.009 mg/kg.

### 2.3. Health Risk Assessment

The evaluation of the health risks associated with consuming mercury-contaminated fish was conducted according to the methodology proposed by the World Health Organization [25] considering the following steps.

#### 2.3.1. Characterization of the Study Population

This stage involved defining the population groups under investigation (i.e., gender and age range) and estimating their respective average weights (in kg) and the average amount of fish consumed daily (in g).

The following population groups were considered: (i) women of childbearing age (from 10 to 49 years); (ii) adult men (≥18 years); (iii) children aged from 5 to 12 years; and (iv) children aged from 2 to 4 years.

The body weight data for each population group were obtained by consulting the Family Budget Survey (POF, 2008), which was organized by the IBGE Automatic Retrieval System (the most recent data available for public consultation). The following body weight averages were used: (i) 50.95 kg for women of childbearing age; (ii) 66.88 kg for adult men; (iii) 27.92 kg for children aged from 5 to 12 years; and (iv) 14.49 kg for children aged from 2 to 4 years.

The estimate of fish consumption by the population of the Amazon was based on a report on fish consumption in the Amazon region of Brazil. The report indicated an average per capita consumption of approximately 100 g of fish per day in urban areas [12].

#### 2.3.2. Estimate of Daily Intake of Mercury

The following assumptions were made: (i) 100% of the mercury detected in the fish samples was in the chemical form of methylmercury (MeHg) and (ii) approximately 80% of the amount of mercury ingested in food was absorbed by the human gastrointestinal tract (absorption rate).

#### 2.3.3. Calculation of the Risk Ratio

The risk ratio (RR) indicated the potential health damage caused by consuming contaminated fish. The calculation was performed by dividing the average amount absorbed by the human body (i.e., 80% of the ingested dose) by the reference dose. For this study, the safe daily intake dose of 0.1 µg MeHg/kg body weight/day proposed by the Environmental Protection Agency (U.S. EPA) [26] was considered as a reference.

When the RR < 1, the absorbed dose of mercury was lower than the reference dose considered. Consequently, the risk of becoming sick was low. On the other hand, when the RR ≥ 1, the absorbed dose of mercury exceeded the reference dose considered, and the risk of becoming sick due to exposure to mercury should be considered. The higher the RR, the greater the potential risk of harm to the health of the population.

#### 2.3.4. Maximum Safe Consumption (MSC) Indication of Fish 

In conclusion of the health risk assessment, the maximum safe consumption (MSC) value of fish was defined for the four population groups by multiplying the reference dose by the average body weights and was presented in grams/day. Following this, the product of this multiplication was divided by the average total mercury concentration (µg/g) detected in different fish species. As there were regional preferences in fish consumption, as well as different levels of mercury accumulation depending on the diet of each fish species [27,28], consumption was standardized to an average of 50% carnivorous fish species and 50% noncarnivorous fish species.

### 2.4. Statistical Analysis

In order to explore factors associated with levels of mercury contamination in fish ≥ 0.5 µg/g in the studied locations, a Poisson regression was performed using the prevalence ratio (PR) as the measure of association and considering a 95% confidence interval. After the initial raw analysis, the variables that demonstrated a level of significance (*p*-value) < 0.05 remained in the final model. The data were analyzed using Statistical Package for the Social Sciences (SPSS), version 9.0 (SPSS, Chicago Inc.: Chicago, IL, USA).

## 3. Results

A total of 1010 fish specimens were sampled, belonging to 80 distinct species distributed across four trophic levels: herbivores, detritivores, omnivores, and carnivores. Overall, 159 samples presented mercury levels below the detection limit, and 38 presented mercury levels below the quantification limit, totaling 197 samples (19.5%) in which it was not possible to estimate the levels of mercury contamination.

The concentrations of mercury in fish ranged from 0 to 4.73 μg/g, with an average concentration of 0.34 μg/g (standard deviation of 0.56 and median of 0.13 μg/g) (Table 1). A total of 21.3% presented levels equal to or greater than 0.5 µg/g in sampled fish.

Analyzing the different trophic levels overall, 110 herbivorous fish, 130 detritivores, 286 omnivores, and 484 carnivores were sampled. The average concentrations of mercury among noncarnivorous fish (i.e., herbivores, detritivores, and omnivores) and carnivorous fish were 0.092 μg/g (*n* = 526) and 0.603 μg/g (*n* = 484), respectively (Table 1).

Considering the levels of mercury contamination, the risk ratio, and the maximum safe fish consumption based on the state’s geographic division, the results were as follows. In Acre (AC), 78 fish specimens from 25 different species were sampled. The average concentration of mercury was 0.58 μg/g and the median was 0.15 μg/g, with 35.9% of the samples exceeding the safe limit of 0.5 μg/g (Table 1). The analysis of the risk associated with fish consumption revealed that the daily intake of mercury exceeded the reference dose recommended by the U.S. EPA (0.1 μg/kg bw/day) in all population groups analyzed (Table 2). In summary, the potential intake of mercury ranged from 7 to 31 times higher than the reference dose recommended by the U.S. EPA. Analyzing the population groups most vulnerable to the effects of mercury, women of childbearing age may be ingesting approximately nine times more mercury than the recommended safe dose, whereas children aged from two to four years may be ingesting up to thirty-one times more Hg (Table 2). In the state, the most mercury-contaminated fish were Cachorra (average: 1.45 μg/g), Filhote (average: 2.07 μg/g), and Dourada (average: 3.57 μg/g). On the other hand, Pacú, Pirapitinga, and Tambaqui can be consumed freely by all analyzed population groups since they had mercury levels close to zero (i.e., lower than 0.0005 μg/g and, therefore, undetectable by the analytical method). Furthermore, Tilápia, Jatuarana, Aracú Cabeça Gorda, and Acará can be safely consumed by adult men and women of childbearing age in quantities ranging from 103 to 418 g/day (Table 3).

A total of 114 fish specimens from 27 distinct species were sampled in Amapá (AP). The average concentration of mercury was 0.18 μg/g, the median was 0.08 μg/g, and 11.4% of the samples had mercury levels higher than 0.5 μg/g (Table 1). The analysis of the risk associated with fish consumption revealed that the daily intake of mercury exceeded the reference dose recommended by the U.S. EPA (0.1 μg/kg bw/day) in all population groups. Mercury intake ranged from 1.7 to 8 times more than the reference dose. Among the population groups most vulnerable to the effects of mercury, women of childbearing age ingested approximately four times more mercury than the recommended dose, and children aged from two to four years ingested eight times more (Table 2). The most mercury-contaminated fish were Uéua (average: 0.49 μg/g), Traíra (average: 0.53 μg/g), and Tucunaré (average: 0.84 μg/g). On the other hand, Acari, Aracú Cabeça Gorda, Jatuarana, Pacú, and Pirapitinga can be freely consumed by all population groups analyzed since they had mercury levels close to zero (i.e., below 0.0005 μg/g and, therefore, undetectable by the analytical method). Furthermore, Tambaqui, Aracú, and Pescada Amarela can be safely consumed by all population groups in quantities ranging from 120 to 1,114 g/day (Table 4).

A total of 262 fish specimens from 34 different species were analyzed in Amazonas (AM). The average concentration of mercury was 0.34 μg/g, and the median was 0.14 μg/g, with 22.5% of the samples exceeding the safe limit of mercury (Table 1). The analysis of the risk associated with fish consumption revealed that the daily intake of mercury exceeded the reference dose in all analyzed population groups (Table 2). In summary, the intake of mercury ranged from 5 to 21 times higher than the reference dose. Women of childbearing age ingested approximately six times the recommended dose, whereas the group of children aged from two to four years ingested twenty-one times more mercury than the recommended dose. The most mercury-contaminated fish were Apapá (average: 1.49 μg/g), Pirapucu (average: 1.61 μg/g), and Filhote (average: 1.70 μg/g). The fish with the lowest concentrations of mercury were Jundiá, Acari, Pacú, Pirapitinga, and Tambaqui. These species showed average levels of mercury below 0.03 μg/g and, therefore, can be consumed in quantities ranging from 107 to 668 g/day by women of childbearing age, children aged from 5 to 12 years, and adult men (Table 5).

A total of 393 fish specimens were collected from 47 distinct species in Pará (PA). The average concentration of mercury was 0.27 μg/g, the median was 0.1 μg/g, and 15.8% of the collected fish had mercury levels above 0.5 μg/g (Table 1). The analysis of the risk associated with fish consumption revealed that mercury intake could be from 3 to 16 times higher than the reference dose. Women of childbearing age ingested approximately four times the recommended dose of mercury, and children aged from two to four years old ingested fifteen times more. The most mercury-contaminated fish were Pirarara (average: 0.92 μg/g), Jaú (average: 0.95 μg/g), and Barbado (average: 1.58 μg/g). The Pacú Branco, Pirapitinga, and Pratiqueira, in turn, can be consumed freely by all population groups analyzed since they had mercury levels close to zero (i.e., lower than 0.0005 μg/g and, therefore, undetectable by the analytical method). Furthermore, the Pacú Manteiga, Tambaqui, Pacú, Tainha, and Aracú can be safely consumed by women of childbearing age, children aged from 5 to 12, and adult men in quantities ranging from 126 to 2229 g/day (Table 6).

A total of 88 fish samples from 28 different species were analyzed in Rondônia (RO). The average concentration of mercury was 0.45 μg/g, and the median was 0.16 μg/g, with 26.1% of the fish having mercury levels above 0.5 μg/g (Table 1). The analysis of the risk associated with fish consumption revealed that daily intake of mercury exceeded the reference dose from 6 to 27 times in all population groups analyzed (Table 2). Women of childbearing age ingested approximately eight times more mercury than men, and children aged from two to four years ingest twenty-seven times more mercury than adults. The most mercury-contaminated fish were Dourada (average: 1.81 μg/g), Filhote (average: 1.84 μg/g), and Babão (average: 2.87 μg/g). However, Acará, Bacu, and Pirapitinga can be consumed freely by all analyzed population groups, as they had mercury levels close to zero. Furthermore, Pacú can be safely consumed by women of childbearing age and adult men in quantities ranging from 137 to 180 g/day (Table 7).

A total of 75 fish specimens from 27 different species were collected in Roraima (RR). The average concentration of mercury was 0.55 μg/g, and the median was 0.41 μg/g, with 40% of the fish exceeding the safety limit (Table 1). The analysis of the risk associated with fish consumption revealed that the daily intake of mercury exceeded the reference dose in all population groups analyzed (Table 2), ranging from 6 to 27 times higher. Women of childbearing age ingested approximately eight times and children aged from two to four years ingest twenty-seven times more mercury than the recommended dose. The most mercury-contaminated fish were Pindirá (average: 1.07 μg/g), Filhote (average: 1.14 μg/g), Piracatinga (average: 1.49 μg/g), Barba Chata (average: 2.00 μg/g), and Coroataí (average: 2.13 μg/g). The fish with the lowest mercury concentrations were Pacú Maria Antônia, Aracú Flamengo, Pacú Meião, Pacú, and Jaraqui Escama Grossa. These species showed average mercury levels below 0.05 μg/g and, therefore, can be consumed in quantities ranging from 125 to 418 g/day by women of childbearing age and adult men (Table 8).

Finally, the Poisson regression analysis revealed that the prevalence of mercury contamination ≥ 0.5 µg/g was approximately 14 times higher in carnivorous fish than in noncarnivorous fish (PR 13.8; 95% CI 8.4–22.5). The prevalence of mercury contamination ≥ 0.5 µg/g was approximately four times higher in Roraima (PR 3.9; 95% CI 2.3–6.7) and Acre (PR 3.9; 95% CI 2.3–6.6), three times higher in Rondônia (PR 3.1; 95% CI 1.8–5.6) and Amazonas (PR 2.9; 95% CI 1.7–5.0), and two times higher in Pará (PR 1.9; 95% CI 1.1–3.1) compared to Amapá (Table 9).

Additional data on the fish contamination according to the studied municipalities, including the mean and median of mercury levels the trophic level of the sampled species, and the prevalence of exposure above 0.5µg/g can be seen in Table 10.

## 4. Discussion

Although many authors have conducted investigations dedicated to analyzing the levels of mercury contamination in different areas of the Amazon at different times, this is the first study that carried out a risk assessment to human health attributed to the consumption of fish contaminated with mercury in urban areas. The samples were collected from places where most of the fish were commercialized in the 17 municipalities assessed in six Brazilian states.

Despite the numerous benefits associated with regular fish consumption, such as reducing blood cholesterol levels, decreasing the risk of myocardial infarction, and improving cognitive development, the increasing contamination of fish with methylmercury represents an important warning signal that authorities should not neglect. Public policies must consider the significance of the fishing industry and its professionals (fishermen), who are also greatly impacted by the increasing contamination. Currently, there are over 350,000 registered professional fishermen in the Secretariat of Aquaculture and Fisheries (2022), with an estimated total fish production of approximately 200,000 tons per year [20]. The estimated economic impact of inland fishing in Brazil is USD 828 million [29], with most of these fisheries occurring in the Amazon region.

Our analysis revealed that more than one-fifth (21.3%) of the fish sold in urban centers, which reach the tables of families in these regions, had mercury levels above the safe limits established by the Food and Agriculture Organization of the United Nations (FAO/WHO) [30] and the Brazilian Health Surveillance Agency (ANVISA) [31] (i.e., ≥ 0.5 µg/g).

The analysis by state revealed that Acre had the highest levels of mercury contamination (average = 0.58 µg/g), whereas the highest prevalence of contamination (i.e., fish with mercury levels ≥0.5 µg/g) was detected in the state of Roraima (40%). On the other hand, Amapá had the lowest levels of contamination (average = 0.18 µg/g), as well as the lowest prevalence of fish with mercury levels above 0.5 µg/g (11.40%).

When considering contamination prevalence ≥0.5 µg/g, the situation becomes slightly different, with Roraima surpassing Acre and taking the first position. The order of contamination prevalence was as follows: Amapá (11%) < Pará (16%) < Amazonas (22%) < Rondônia (26%) < Acre (36%) < Roraima (40%). Our results revealed that the most serious situations of mercury contamination in fish were concentrated in Roraima, Rondônia, and Acre. As widely reported by various authors [28,32,33,34], the increase in illegal gold-mining activities in Roraima and Rondônia is directly related to the high levels of mercury detected in the fish from these regions.

The comparative analysis based on the average levels of mercury in fish samples, as well as the Poisson regression analysis, indicated an increasing contamination in the municipalities that made up the states according to the following ranking: Amapá (0.18 µg/g) < Pará (0.27 µg/g) < Amazonas (0.34 µg/g) < Rondônia (0.45 µg/g) < Roraima (0.55 µg/g) < Acre (0.58 µg/g). It was reported that contamination levels were fourteen times higher in carnivorous fish compared to noncarnivorous fish and approximately four times higher in Roraima and Acre, three times higher in Rondônia and Amazonas, and two times higher in Pará when compared to Amapá.

The results obtained in Acre are intriguing and should be interpreted with caution. Although there are few reports and records of gold-mining activity in the region, other studies [35,36,37,38] have indicated the presence of high levels of mercury in fish samples and other food products. This suggests that the availability of mercury in the region may be influenced by other anthropogenic sources of mercury emissions. Moreover, a significant portion of the fish sold in Rio Branco (the state capital), especially in the Elias Mansou Market, is sourced from the municipalities of Boca do Acre and Porto Velho, which are known to be affected by gold mining.

Despite the differences observed in the average levels of mercury or the prevalence of contamination above 0.5 µg/g, the analysis of the risk attributable to fish consumption according to the state revealed that daily mercury intake exceeded the reference dose recommended by the U.S. EPA (0.1 μg/kg bw/day) in all population groups analyzed and in all states of the Amazon region studied. However, the risks associated with the consumption of contaminated fish are diverse and must be taken into account to formulate appropriate nutritional guidelines for safe fish consumption by the local community. Accordingly, we prioritized the three states with the most concerning results.

For Acre, the ingestion of mercury through consumption of contaminated fish was found to be from 7 to 31 times higher than the recommended safe dose. In the sampled municipalities, Cachorra, Filhote, and Dourada should be avoided or consumed exceptionally. On the other hand, Pacú, Pirapitinga, and Tambaqui can be consumed freely. Moreover, Acará, Aracú Cabeça Gorda, Jatuarana, and Tilápia can be safely consumed by adult men and women of childbearing age in quantities ranging from 103 to 418 g/day. However, these fish are not recommended for children.

In Roraima, mercury intake was found to be from 6 to 27 times higher than the dose recommended by the U.S. EPA. In the sampled municipalities, it is recommended to avoid the consumption of Barba Chata, Coroataí, Pindirá, and Piracatinga for all population groups. On the other hand, Aracú Flamengo, Pacú Maria Antônia, Pacú Meião, Pacú, and Jaraqui Escama Grossa can be consumed by women of childbearing age and adult men in quantities ranging from 125 to 418 g/day. However, children should consume these fish in moderation, not exceeding 44, 36, 38, 91, and 44 g/day, respectively, for the age group from 2 to 4 years and 85, 69, 73, 175, and 86 g/day, respectively, for the age group from 5 to 12 years.

In Rondônia, the results revealed that mercury intake varied at levels from 6 to 27 times higher than the recommended safe dose. In these municipalities, it is recommended to restrict the consumption of Babão, Dourada, and Filhote. On the other hand, Acará, Bacu, and Pirapitinga can be consumed freely by all analyzed population groups. Furthermore, Pacú can be safely consumed by women of childbearing age and adult men in quantities ranging from 137 to 180 g/day.

Without losing sight of the illustrative findings of this investigation, it is important to consider some limitations inherent in the ecological study design. Although 1010 fish specimens, representing 80 distinct species distributed across four trophic levels and originating from at least six river basins in the Brazilian Amazon, were included, the analyzed data do not have the capacity to represent the entire diversity of fish available for human consumption in the region. Another point to be considered is the difficulty of collecting samples during different seasons of the year, considering the rainy and dry periods in the Amazon and their influence on the availability of fish and other food. Therefore, it is possible that, despite conservative estimates, our findings are subject to selection bias and may not reveal the true impact of mercury exposure for the majority of the current population living in urban centers of the Amazon.

The estimated risk factors indicated that strict dietary guidelines are necessary for safe consumption of fish. Comparing the doses of mercury intake among the states, we observed that the risks were varied and higher with the consumption of carnivorous fish species, especially in Acre, Roraima, and Rondônia.

According to safety parameters established by the U.S. Environmental Protection Agency (U.S. EPA), in practically all the locations studied, the risk of becoming sick due to consuming fish contaminated with methylmercury was high, especially among children.

Meanwhile, it is worth acknowledging that the safe intake dose considered by the U.S. EPA was estimated from data produced in longitudinal studies conducted in the Faroe Islands, Denmark. That is, this parameter was estimated based on observations of populations living in another part of the planet with distinct dietary habits and subject to diverse conditions from those experienced in the Amazon region, both from a socioeconomic point of view, as well as from a cultural and access to health services perspective.

The use of this reference parameter may have produced distorted results (with attenuated risk estimates) because other risk factors in the Amazonian ecosystem may increase human exposure to mercury. Some studies have indicated that, in addition to the presence of natural mercury in the soil of the Amazon [27,39,40], the expansion of agribusiness, the construction of dams and hydroelectric plants, burning, and other activities that promote deforestation significantly alter the biogeochemical cycle of mercury in the environment [4]. This alteration favors the entry of methylmercury into the food chain, thus increasing human exposure and the consequent health risks of contact with this environmental contaminant. These anthropogenic activities in combination with illegal gold mining and indiscriminate use of mercury produce a unique risk situation for the local population.

In addition to the study limitations pointed out previously, it is essential to say that other physical, chemical, and biological parameters can also interfere with the mercury bioaccumulation process and, consequently, with the mercury concentration present in fish muscle tissue. On the one hand, the most critical parameter is the amount of different mercury species bioavailable in the aquatic system, which are closely related to mercury-emitting sources, notably those linked to gold-mining activities in the Amazon region and to the water’s physical-chemical characteristics (i.e., pH, temperature, ions dissolved, etc.). On the other hand, parameters related to fish characteristics such as sex, weight, length, growth rate, and age can also explain the mercury concentrations detected in this investigation [41,42]. Unfortunately, we could not consider these parameters in the interpretation of mercury levels detected in fish samples collected for this study; therefore, this can be considered a limitation as well.

It is worth reminding that another important limitation concerns the different mercury species present in the muscle of the studied fish. Although it is reasonable to assume that all mercury present in the analyzed samples is in the methylmercury form, it is important to clarify that about 15% of the mercury present in fish may be in inorganic form, as has been described in several studies [27,43,44,45,46,47]. In order to attenuate this limitation and to prevent bias in the results interpretation, we assumed that only 80% of the mercury available in the fish muscle tissue was absorbed by the human gastrointestinal tract. Finally, unlike methylmercury, ingestion of inorganic forms of mercury seems not to represent a relevant risk to public health.

## 5. Conclusions

It becomes evident that the development of longitudinal studies involving different population groups in the Amazon (including indigenous peoples, riverine communities, and quilombolas, as well as those living in urban centers) is especially important. Only a long-term study can lead to more accurate estimates of the risks associated with fish consumption, as well as safe doses of mercury intake for the Amazonian population.

Conversely, the strengths of this study include the geographic scope of the fish collection points included in the risk analyses, the prevalence ratios employed in the multivariate analyses, the methodological rigor used in the collection of fish samples, and the analyses of mercury levels being carried out in national reference laboratories, as well as the assumption that only 80% of the amount of mercury ingested in food was absorbed by the human gastrointestinal tract.

Therefore, we believe that, together, our findings establish a solid foundation for the planning of strategic interventions, as they provide relevant information to guide the safe consumption of fish in the study area and contribute robust scientific evidence to clarify a pressing issue in the field of national public health.

## Figures and Tables

**Table 1:** Levels of mercury detected in fish samples acquired from 17 localities in the Amazon Basin, Brazil, 2021–2022.

State	N	Number of Species	Mean Hg μg/g (D.P *)	Median Hg	Min–Max Hg	Mean Hg μg/g Carnivorous (n)	Mean Hg μg/g Noncarnivorous (n)	% ≥0.5 μg/g
Acre	78	25	0.58 (0.97)	0.15	0.00–4.64	1.06 (40)	0.08 (38)	36
Amapá	114	27	0.18 (0.25)	0.08	0.00–1.24	0.27 (74)	0.02 (40)	11
Amazonas	262	34	0.34 (0.49)	0.14	0.00–3.22	0.67 (108)	0.11 (154)	22
Pará	393	47	0.27 (0.43)	0.1	0.00–3.50	0.48 (183)	0.08 (210)	16
Rondônia	88	28	0.45 (0.80)	0.16	0.00–4.73	0.84 (40)	0.13 (48)	26
Roraima	75	27	0.55 (0.65)	0.41	0.00–3.55	0.87 (43)	0.12 (32)	40
Amazon Region	1010	80	0.34 (0.56)	0.13	0.00–4.73	0.60 (488)	0.09 (522)	21

* Standard deviation.

**Table 2:** Attributable risk ratio for consumption of mercury-contaminated fish according to the reference dose recommended by the U.S. EPA by Federal Units and population groups analyzed in the Amazon Basin, Brazil, 2021–2022.

State	Population Group	Ingested Dose (µg/kg bw/day)	Absorbed Dose ~80% (µg/kg bw/day)	Risk Ratio (U.S. EPA)
Acre	Adult men	0.85	0.68	7
	Women of Childbearing Age	1.12	0.9	9
	Children aged from 5 to 12 years	2.04	1.63	16
	Children aged from 2 to 4 years	3.94	3.15	31
	Adult men	0.22	0.17	2
Amapá	Women of Childbearing Age	0.28	0.23	2
	Children aged from 5 to 12 years	0.52	0.42	4
	Children aged from 2 to 4 years	1.00	0.80	8
	Adult men	0.58	0.47	5
Amazonas	Women of Childbearing Age	0.76	0.61	6
	Children aged from 5 to 12 years	1.40	1.12	11
	Children aged from 2 to 4 years	2.69	2.15	21
	Adult men	0.42	0.34	3
Pará	Women of Childbearing Age	0.56	0.45	4
	Children aged from 5 to 12 years	1.02	0.81	8
	Children aged from 2 to 4 years	1.96	1.57	16
	Adult men	0.72	0.58	6
Rondônia	Women of Childbearing Age	0.95	0.76	7
	Children aged from 5 to 12 years	1.73	1.39	14
	Children aged from 2 to 4 years	3.34	2.67	27
	Adult men	0.74	0.59	6
Roraima	Women of Childbearing Age	0.97	0.77	8
	Children aged from 5 to 12 years	1.76	1.41	14
	Children aged from 2 to 4 years	3.40	2.72	27

**Table 3:** Characterization of fish acquired in the state of Acre and calculation of the maximum safe consumption (MSC) in grams in the Amazon Basin, Brazil, 2021–2022.

Fish Characterization							MSC (g/day)			
Common Name	Scientific Name	N	Mean Hg μg/g	Trophic Level	Mean Length (cm)	Mean Weight (g)	Adult Men	Women	Children Aged from 5 to 12 Years	Children Aged from 2 to 4 Years
Acará	Cichlidae	3	0.05	Omnivorous	21.16	213.33	136.49	104	57	29
Acari	Loricariidae	1	0.13	Detritivorous	39.00	465.00	53.08	40	22	11
Aracu Cabeça Gorda	*Leporinus* spp.	6	0.04	Omnivorous	36.48	848.33	171.49	130	71	37
Aracu Flamengo	*Leporinus fasciatus*	3	0.37	Omnivorous	31.50	395.00	18.08	14	7	4
Bico de Pato	*Sorubim lima*	2	0.86	Carnivorous	36.50	295.00	7.75	6	3	2
Branquinha	*Psectrogaster* sp.	2	0.11	Detritivorous	24.15	165.00	61.93	47	26	13
Cachorra	Cynodontidae	4	1.45	Carnivorous	42.50	730.00	4.62	3	2	1
Curimatã	*Prochilodus nigricans*	6	0.09	Detritivorous	51.17	1314.17	74.31	56	31	16
Dourada	*Brachyplatystoma rousseauxii*	3	3.57	Carnivorous	N.D.	N.D.	1.87	1	1	0
Filhote	*Brachyplatystoma filamentosum*	3	2.07	Carnivorous	N.D.	N.D.	3.24	2	1	1
Jatuarana	*Brycon* sp.	3	0.02	Omnivorous	43.35	1395.00	393.41	300	164	85
Jundiá	Pimelodiae	4	1.19	Carnivorous	74.00	1595.00	5.62	4	2	1
Mandi	*Pimelodus blochii*	1	0.17	Omnivorous	23.00	115.00	38.88	30	16	8
Pacú	*Myleus* sp.	3	0	Herbivorous	22.85	290.00	N.R.	N.R.	N.R.	N.R.
Pescada	*Plagioscion squamossimus*	3	0.94	Carnivorous	31.00	290.00	7.15	5	3	1
Piabinha	Characidae	3	0.09	Omnivorous	19.33	108.33	71.91	55	30	15
Pintadinho	*Calophysus macropterus*	3	1.12	Carnivorous	N.D.	593.33	5.95	4	2	1
Pirapitinga	*Piaractus brachypomus*	1	0	Omnivorous	60.00	3510.00	N.R.	N.R.	N.R.	N.R.
Pirarucu	*Arapaima gigas*	3	0.69	Carnivorous	N.D.	N.D.	9.75	7	4	2
Surubim	*Pseudoplatystoma* sp.	6	0.64	Carnivorous	47.92	6290.00	10.50	8	4.	2
Tambaqui	*Colossoma macropomum*	3	0	Omnivorous	61.33	4153.00	N.R.	N.R.	N.R.	N.R.
Tamoatá	*Hoplosternum littorale*	3	0.20	Omnivorous	17.17	75.00	33	25	14	7
Tilápia	*Oreochromis* sp.	3	0.02	Omnivorous	37.43	1050.00	418	318	174	90
Traíra	*Hoplias malabaricus*	3	0.08	Carnivorous	39.67	608.33	82	63	34	18
Tucunaré	*Cichla* sp.	3	0.21	Carnivorous	33.67	505.00	32	24	13	7

N.D.—no date; N.R.—no restriction for consumption.

**Table 4:** Characterization of fish acquired from the state of Amapá and calculation of the maximum safe consumption (MSC) in grams in the Amazon Basin, Brazil, 2021–2022.

Fish Characterization							MSC (g/day)			
Common Name	Scientific Name	N	Mean Hg μg/g	Trophic Level	Mean Length (cm)	Mean Weight (g)	Adult Men	Women	Children Aged from 5 to 12 Years	Children Aged from 2 to 4 Years
Acará	Cichlidae	3	0.04	Omnivorous	7.33	115	167.20	127	70	36
Acari	Loricariidae	7	0	Detritivorous	44.8	874.71	N.R.	N.R.	N.R.	N.R.
Anujá	*Trachelyopterus galeatus*	3	0.164	Omnivorous	7.33	95	40.78	31	17	9
Apapá	*Pellona* sp.	5	0.254	Carnivorous	51.8	1570.8	26.33	20	11	6
Aracu	*Schizodon fasciatus*	3	0.01	Herbivorous	31.5	382.66	668.80	509	279	145
Aracu Cabeça Gorda	*Leporinus* spp.	3	0	Omnivorous	35.5	608	N.R.	N.R.	N.R.	N.R.
Arraia	*Potamotrygon* sp.	1	0.338	Carnivorous	N.D.	N.D.	19.79	15	8	4
Dourada	*Brachyplatystoma rousseauxii*	6	0.155	Carnivorous	82.33	3425.16	43.15	33	18	9
Filhote	*Brachyplatystoma filamentosum*	5	0.227	Carnivorous	114.33	17858	29.46	22	12	6
Gurijuba	*Sciades parkeri*	3	0.029	Carnivorous	94.33	7012	230	176	96	50
Jacundá	*Crenicichla* sp.	1	0.143	Carnivorous	12.5	375	47	35	19	10
Jatuarana	*Brycon* sp.	3	0	Omnivorous	41	1216	N.R.	N.R.	N.R.	N.R.
Jeju	*Hoplerythrinus unitaeniatus*	3	0.121	Carnivorous	10.17	236.6	55	42	23	12
Mapará	*Hypophthalmus sp.*	3	0.02	Herbivorous	42.67	458.66	334	255	139	72
Pacu	*Myleus* sp., *Myloplus* sp.	2	0	Herbivorous	36.5	769.5	N.R.	N.R.	N.R.	N.R.
Pescada	*Plagioscion squamossimus*	10	0.2604	Carnivorous	63.35	2716.8	26	19	11	5
Pescada Amarela	*Cynoscion acoupa*	7	0.012	Carnivorous	70.36	2985.14	557	424	233	121
Piramutaba	*Brachyplatystoma vaillantii*	4	0.054	Carnivorous	67.5	2025	124	94	52	27
Piranha	*Serrasalmidae*	5	0.254	Carnivorous	13.9	210	26	20	11	6
Pirapitinga	*Piaractus brachypomus*	4	0	Omnivorous	63.88	4898.5	N.R.	N.R.	N.R.	N.R.
Robalo	*Centropomus undecimalis*	3	0.04	Carnivorous	55.5	1396.66	167	127	70	36
Surubim	*Pseudoplatystoma* sp.	3	0.165	Carnivorous	68.67	2593.33	40	31	17	9
Tambaqui	*Colossoma macropomum*	6	0.006	Omnivorous	62.17	4436.8	1115	849	465	241
Tamoatá	*Hoplosternum littorale*	3	0.058	Omnivorous	8.17	151.66	115	88	48	25
Traíra	*Hoplias malabaricus*	12	0.527	Carnivorous	58.17	3867.58	13	10	5	3
Tucunaré	*Cichla* sp.	4	0.844	Carnivorous	54.67	2736	8	6	3	2
Uéua	*Acestrorhynchus falcirostris*	2	0.495	Carnivorous	10.5	157.5	13	10	6	3

N.D.—no date; N.R.—unrestricted consumption.

**Table 5:** Characterization of fish acquired from the state of Amazonas and calculation of the maximum safe consumption (MSC) in grams in the Amazon Basin, Brazil, 2021–2022.

Fish Characterization							MSC (g/day)			
Common Name	Scientific Name	N	Mean Hg μg/g	Trophic Level	Mean Length (cm)	Mean Weight (g)	Adult Men	Women	Children Aged from 5 to 12 Years	Children Aged from 2 to 4 Years
Acará	Cichlidae	8	0.149	Omnivorous	21.56	187.25	44.89	34	19	10
Acará-açu	*Astronotus* sp.	3	0.069	Omnivorous	20.93	203.66	96.93	74	40	21
Acari	Loricariidae	9	0.012	Detritivorous	37.08	511.67	557.33	424	233	121
Apapá	*Pellona* sp.	3	1.492	Carnivorous	56	2166.67	4.48	3	2	1
Aracu	*Schizodon fasciatus*	5	0.078	Herbivorous	27.6	324	85.74	65	36	18
Aracu Cabeça Gorda	*Leporinus* spp.	10	0.092	Omnivorous	34	512.86	72.70	55	30	16
Aracu Flamengo	*Leporinus fasciatus*	5	0.099	Omnivorous	29.28	266	67.56	51	28	15
Aruanã	*Osteoglossum bicirrhosum*	10	0.3713	Carnivorous	60.55	1453.78	18.01	14	7	4
Branquinha	*Psectrogaster* sp.	6	0.0865	Detritivorous	23.38	178	77.32	59	32	17
Cachorra	*Cynodontidae*	5	0.6288	Carnivorous	48.7	877.33	10.64	8	4	2
Charuto	*Hemiodus* sp.	7	0.1681	Omnivorous	25.62	218.43	39.79	30	16	9
Cuiu	*Oxydoras niger*	6	0.177	Omnivorous	51.78	1535.67	37.79	29	16	8
Curimatã	*Prochilodus nigricans*	9	0.043	Detritivorous	28.78	1549.89	155.53	118	65	34
Filhote	*Brachyplatystoma filamentosum*	4	1.702	Carnivorous	80	6601.67	3.93	3	1	1
Jacundá	*Crenicichla* sp.	3	0.4116	Carnivorous	36.33	495	16.25	12	7	3
Jaraqui	*Semaprochilodus* sp.	12	0.1	Detritivorous	25.04	265.33	66.88	51	28	14
Jatuarana	*Brycon* sp.	19	0.071	Omnivorous	31.54	592.79	94.20	72	39	20
Jeju	*Hoplerythrinus unitaeniatus*	3	0.24	Carnivorous	28.67	331.33	27.87	21	12	6
Jundiá	*Pimelodiae*	1	0.01	Carnivorous	50	1255	668.80	509	279	145
Mandi	*Pimelodus blochii*	1	0.783	Omnivorous	21	500	8.54	6	3	2
Mandubé	*Auchenipteridae*	1	0.783	Carnivorous	46	N.D.	8.54	6	3	2
Pacú	*Myleus* sp. *Mylossoma* sp.	13	0.016	Herbivorous	19.75	205.09	418.00	318	174	9
Pescada	*Plagioscion squamossimus*	4	0.799	Carnivorous	37.5	656.67	8.37	6	3	2
Pirandira	*Hydrolycus scomberoides*	4	0.974	Carnivorous	37.75	648.75	6.87	5	3	1
Piranha	*Serrasalmidae*	21	0.762	Carnivorous	24.7	433.72	8.78	7	4	2
Pirapitinga	*Piaractus brachypomus*	7	0.0194	Omnivorous	39.9	1552.14	344.74	263	144	75
Pirapucu	*Cynodontidae*	3	1.609	Carnivorous	45	477.5	4.16	3	2	1
Pirarara	*Phractocephalus hemiolipterus*	7	0.724	Omnivorous	55	4247.5	9.24	7	4	2
Pirarucu	*Arapaima gigas*	4	0.287	Carnivorous	35.33	1233.33	23.30	18	10	5
Sardinha	*Triportheus* sp.	12	0.129	Omnivorous	23.75	137	51.84	39	22	11
Surubim	*Pseudoplatystoma* sp.	7	0.652	Carnivorous	68.42	2259	10.26	8	4	2
Tambaqui	*Colossoma macropomum*	15	0.026	Omnivorous	52.06	4214	257.23	196	107	56
Traíra	*Hoplias malabaricus*	12	0.4215	Carnivorous	39.87	695.91	15.87	12	7	3
Tucunaré	*Cichla* sp.	23	0.567	Carnivorous	37.81	900.04	11.80	9	5	2

**Table 6:** Characterization of fish acquired from the state of Pará and calculation of the maximum safe consumption (MSC) in grams in the Amazon Basin, Brazil, 2021–2022.

Fish Characterization							MSC (g/day)			
Common Name	Scientific Name	N	Mean Hg μg/g	Trophic Level	Mean Length (cm)	Mean Weight (g)	Adult Men	Women	Children Aged from 5 to 12 Years	Children Aged from 2 to 4 Years
Acará-açu	*Astronotus* sp.	3	0.067	Omnivorous	23.83	311.67	99.82	76	42	22
Acaratinga	*Geophagus* sp.	6	0.058	Omnivorous	24.72	250.50	115.31	88	48	25
Acari	Loricariidae	16	0.032	Detritivorous	33.08	398.63	209.00	159	87	45
Apapá	*Pellona* sp.	10	0.202	Carnivorous	39.60	476.80	33.11	25	14	7
Aracu	*Schizodon fasciatus*	12	0.022	Herbivorous	33.27	311.17	304.00	231	127	66
Aracu Cabeça Gorda	*Leporinus* spp.	6	0.054	Omnivorous	42.22	1134.00	123.85	94	52	27
Aracu Flamengo	*Leporinus fasciatus*	7	0.216	Omnivorous	34.42	293.57	30.96	23	13	7
Arraia	*Potamotrygon* sp.	1	0.624	Carnivorous	N.D.	N.D.	10.72	8	4	2
Barbado	*Pinirampus pirinampu*	6	1.584	Carnivorous	67.87	2910.00	4.22	3	2	1
Bico de Pato	*Sorubim lima*	3	0.255	Carnivorous	55.67	900.00	26.23	20	11	6
Branquinha	*Psectrogaster* sp.	8	0.0503	Detritivorous	26.58	202.25	132.96	101	55	29
Cação	*Carcharhinus* sp.	3	0.304	Carnivorous	N.D.	4800.00	22.00	17	9	5
Cachorra	Cynodontidae	9	0.885	Carnivorous	59.50	2917.78	7.56	6	3	2
Charuto	*Hemiodus* sp.	8	0.032	Omnivorous	19.00	67.13	209.00	159	87	45
Corvina	Sciaenidae	6	0.424	Carnivorous	68.67	2919.17	15.77	12	6	3
Curimatã	*Prochilodus nigricans*	16	0.071	Detritivorous	34.61	757.88	94.20	72	39	20
Dourada	*Brachyplatystoma rousseauxii*	13	0.475	Carnivorous	83.19	4831.92	14.08	11	6	3
Fidalgo	*Ageneiosus* sp.	4	0.457	Carnivorous	57.75	2280.00	14.63	11	6	3
Filhote	*Brachyplatystoma filamentosum*	14	0.598	Carnivorous	91.35	13703.21	11.18	8	5	2
Gó	*Macrodon ancylodon*	3	0.032	Carnivorous	N.D.	8366.67	209.00	159	87	45
Gurijuba	*Sciades parkerii*	3	0.126	Carnivorous	N.D.	8366.67	53.08	40	22	11
Jaraqui	*Semaprochilodus* sp.	15	0.0641	Detritivorous	3015.47	564.47	104.34	79	43	23
Jatuarana	*Brycon* sp.	18	0.0528	Omnivorous	38.73	1060.44	126.67	96	53	27
Jaú	*Zungaro zungaro*	3	0.954	Carnivorous	82.33	6424.33	7.01	5	3	1
Jiripoca	*Hemisorubim platyrhynchos*	3	0.363	Carnivorous	51.66	1026.67	18.42	14	8	4
Mandi	*Pimelodus blochii*	1	0.635	Omnivorous	43.00	590.00	10.53	8	4	2
Mapará	*Hypophthalmus* sp.	20	0.227	Herbivorous	43.55	427.80	29.46	22	12	6
Pacu	*Myleus* sp. *Mylossoma* sp.	15	0.0104	Herbivorous	33.07	864.73	643.08	490	268	139
Pacu Branco	*Myleus* sp.	3	0	Herbivorous	32.83	764.00	N.R.	N.R.	N.R.	N.R.
Pacu Manteiga	*Mylossoma duriventre*	11	0.003	Herbivorous	26.16	182.27	2229.33	1698	931	48
Pescada	*Plagioscion squamossimus*	19	0.306	Carnivorous	43.54	1179.00	21.86	17	9	5
Pescada Amarela	*Cynoscion acoupa*	3	0.158	Carnivorous	94.00	7196.67	42.33	32	18	9
Piramutaba	*Brachyplatystoma vaillantii*	4	0.1307	Carnivorous	62.25	2011.25	51.17	39	21	11
Piranha	*Serrasalmidae*	18	0.479	Carnivorous	30.37	736.06	13.96	11	6	3
Pirapema	*Megalops atlanticus*	3	0.165	Carnivorous	96.33	6866.67	40.53	31	17	9
Pirapitinga	*Piaractus brachypomus*	7	0	Omnivorous	40.93	1452.57	N.R.	N.R.	N.R.	N.R.
Pirarara	*Phractocephalus hemiolipterus*	5	0.921	Omnivorous	78.40	9918.80	7.26	5	3	1
Pirarucu	*Arapaima gigas*	4	0.391	Carnivorous	115.00	34767.50	17.10	13	7	4
Pratiqueira	*Mugil* sp.	3	0	Detritivorous	31.00	366.67	N.R.	N.R.	N.R.	N.R.
Serra	*Scomberomorus brasiliensis*	3	0.08	Carnivorous	66.66	1366.67	83.60	64	35	18
Surubim	*Pseudoplatystoma* sp.	17	0.471	Carnivorous	63.68	2530.35	14.20	11	6	3
Tainha	*Mugil* sp.	3	0.018	Detritivorous	50.66	1566.67	371.56	283	155	80
Tambaqui	*Colossoma macropomum*	21	0.0098	Omnivorous	56.36	3548.86	682.45	520	285	148
Traíra	*Hoplias malabaricus*	5	0.3818	Carnivorous	54.00	2312.00	17.52	13	7	4
Tamoatá	*Hoplosternum littorale*	6	0.0815	Omnivorous	19.98	145.83	82.06	62	34	18
Tucunaré	*Cichla* sp.	23	0.54	Carnivorous	47.24	1836.65	12.39	9	5	3
Zebra	Pimelodidae	3	0.731	Carnivorous	76.66	4700	9.15	7	4	2

N.D.—no date; N.R.—no restriction for consumption.

**Table 7:** Characterization of fish acquired from the state of Rondônia and calculation of the maximum safe consumption (MSC) in grams in the Amazon Basin, Brazil, 2021–2022.

Fish Characterization							MSC (g/day)			
Common Name	Scientific Name	N	Mean Hg μg/g	Trophic Level	Mean Length (cm)	Mean Weight (g)	Adult Men	Women	Children Aged from 5 to 12 Years	Children Aged from 2 to 4 Years
Acará	Cichlidae	3	0	Omnivorous	19.66	153.33	N.R.	N.R.	N.R.	N.R.
Acará-açu	*Astronotus* sp.	3	0.199	Omnivorous	26.33	467.66	33.61	26	14	7
Aracu	*Schizodon fasciatus*	3	0.1	Herbivorous	31.66	301	66.88	51	28	14
Aracu Flamengo	*Leporinus fasciatus*	3	0.32	Omnivorous	28.66	257	20.90	16	9	4
Babão	*Goslinia platynema*	3	2.87	Carnivorous	72.66	3533.33	2.33	18	1	0
Bacu	*Lithodoras dorsalis*	1	0	Omnivorous	N.D.	N.D.	N.R.	N.R.	N.R.	N.R.
Branquinha	*Psectrogaster* sp.	3	0.08	Detritivorous	28.33	310.33	83.60	64	35	18
Cangati	Auchenipteridae	2	0.055	Carnivorous	21.5	175	121.60	93	51	26
Cuiu	*Oxydoras niger*	2	0.241	Omnivorous	82	5577.5	27.75	21	12	6
Curimatã	*Prochilodus nigricans*	3	0.019	Detritivorous	32.66	542.66	352.00	268	147	76
Dourada	*Brachyplatystoma rousseauxii*	3	1.807	Carnivorous	88	7583	3.70	3	1	1
Filhote	*Brachyplatystoma filamentosum*	3	1.844	Carnivorous	112.5	24333.33	3.63	3	1	1
Jaraqui	*Semaprochilodus* sp.	3	0.115	Detritivorous	27.33	337.33	58.16	44	24	13
Jatuarana	*Brycon* sp.	6	0.0751	Omnivorous	34.33	768.66	89.05	68	37	19
Jundiá	Pimelodidae	1	0.309	Carnivorous	62	2945	21.64	16	9	5
Mandi	*Pimelodus blochii*	3	0.071	Omnivorous	23.66	120	94.20	72	39	20
Pacu	*Myleus* sp. *Mylossoma* sp.	3	0.037	Herbivorous	22.33	346.33	180.76	138	75	39
Pintadinho	*Calophysus macropterus*	1	0.22	Carnivorous	37	355	30.40	23	13	6
Piramutaba	*Brachyplatystoma vaillantii*	1	0.279	Carnivorous	30	235	23.97	18	10	5
Piranha	*Serrasalmidae*	3	0.151	Carnivorous	23.66	461	44.29	34	18	10
Pirapitinga	*Piaractus brachypomus*	3	0	Omnivorous	29.33	642	N.R.	N.R.	N.R.	N.R.
Pirarara	*Phractocephalus hemiolipterus*	1	0.816	Omnivorous	29.33	642	8.20	6	3	2
Pirarucu	*Arapaima gigas*	5	0.323	Carnivorous	102	7950	20.71	16	9	4
Sardinha	*Triportheus* sp.	2	0.199	Omnivorous	17.5	121.5	33.61	26	14	7
Surubim	*Pseudoplatystoma* sp.	13	0.778	Carnivorous	68.92	3716.84	8.60	6	3	2
Tambaqui	*Colossoma macropomum*	4	0.262	Omnivorous	60.5	4954.25	25.53	19	11	5
Traíra	*Hoplias malabaricus*	3	0.144	Carnivorous	36	656	46.44	35	19	10
Tucunaré	*Cichla* sp.	4	0.244	Carnivorous	38.5	911	27.41	21	11	6

N.D.—no date; N.R.—no restriction for consumption.

**Table 8:** Characterization of fish acquired from the state of Roraima and calculation of the maximum safe consumption (MSC) in grams in the Amazon Basin, Brazil, 2021–2022.

Fish Characterization							MSC (g/day)			
Common Name	Scientific Name	N	Mean Hg μg/g	Trophic Level	Mean Length (cm)	Mean Weight (g)	Adult Men	Women	Children Aged from 5 to 12 Years	Children Aged from 2 to 4 Years
Acará-açu	*Astronotus* sp.	4	0.332	Omnivorous	33	626.25	20.14	15	8	4
Aracu Cabeça Gorda	*Leporinus* sp.	1	0.147	Omnivorous	29	253	45.50	35	19	10
Aracu Flamengo	*Leporinus fasciatus*	2	0.033	Omnivorous	27.75	233	202.67	154	84	44
Aracu Mandioca	*Schizodon fasciatus*	2	0.238	Carnivorous	32	480	28.10	21	11	6
Barba Chata	*Pirinampus pirinampu*	3	1.997	Carnivorous	47.3	868.33	3.35	2	1	1
Coroataí	*Platynematichthys notatus*	4	2.131	Detritivorous	51.75	1378	3.14	2	1	1
Curimatã	*Prochilodus nigricans*	3	0.097	Detritivorous	28.07	358.33	68.95	52	29	15
Dourada	*Brachyplatystoma rousseauxii*	2	0.673	Carnivorous	80.5	1280	9.94	7	4	2
Filhote	*Brachyplatystoma filamentosum*	3	1.139	Carnivorous	99	18348.33	5.87	4	2	1
Jandiá	*Leiarius* cf. *mamoratus*	2	0.094	Carnivorous	49.75	1111	71.15	54	30	15
Jaraqui Escama Grossa	*Semaprochilodus insignis*	2	0.0405	Detritivorous	29	380	165.14	126	69	36
Liro	*Hemisorubim platyrhynchos*	1	0.413	Carnivorous	38	354	16.19	12	7	3
Mandi	*Pimelodus blochii*	2	0.423	Omnivorous	21	66.5	15.81	12	7	3
Mandubé	*Ageneiosus inermis*	1	0.539	Carnivorous	39	580	12.41	9	5	3
Mantrinxã	*Brycon falcatus*	12	0.132	Omnivorous	27.93	357.5	50.67	38	21	11
Pacu	*Myloplus* sp.	3	0.0386	Herbivorous	28.67	553.33	173.26	132	72	37
Pacu Maria Antonia	*Myleus* sp.	3	0.016	Herbivorous	20.33	218.33	418.00	318	174	90
Pacu Meião	*Myleus* sp.	2	0.033	Herbivorous	20.25	178	202.67	154	85	44
Pescada	*Plagioscion squamosissimus*	3	0.721	Herbivorous	34.5	591	9.28	7	4	2
Pescado Branca	*Plagioscion squamosissimus*	4	0.512	Herbivorous	37.8	543.75	13.06	10	5	3
Piracatinga	*Calophysus macropterus*	1	1.495	Carnivorous	45.6	770	4.47	3	2	1
Pindirá/Peixe Cachorro	*Hydrolycus scomberoides*	1	1.072	Carnivorous	56.6	2025	6.24	5	3	1
Piranha Petra	*Serrasalmus rhombeus*	2	0.406	Carnivorous	21.75	227	16.47	12	7	3
Surubim	*Pseudoplatystoma* sp.	7	0.649	Carnivorous	53.56	1134.71	10.31	8	4	2
Tucunaré	*Cichla* sp.	4	0.698	Carnivorous	41.02	986.25	9.58	7	4	2
Tucunaré Borboleta	*Cichla monoculus*	1	0.645	Carnivorous	38.1	805	10.37	8	4	2

**Table 9:** Poisson regression considering the response variable as levels of Hg ≥ 0.5 µg/g and the independent variables as trophic level and Federal Unit where fish were acquired in the Brazilian Amazon in 2021–2022.

Variables	PR * Crude	95% CI	*p*-Value	PR Adjusted	95% CI	*p*-Value
Trophic Level						
Noncarnivorous	1.0					
Carnivorous	13.3	8.1–21.8	0.001	13.8	8.4–22.5	0.001
State						
AP	1.0					
PA	1.4	0.8–2.4	0.256	1.9	1.1–3.1	0.025
AM	2.0	1.1–3.5	0.017	2.9	1.7–5.0	0.001
RO	2.3	1.2–4.3	0.009	3.1	1.8–5.6	0.001
AC	3.1	1.7–5.7	0.001	3.9	2.3–6.6	0.001
RR	3.5	2.0–6.3	0.001	3.9	2.3–6.7	0.001

* Prevalence ratio.

**Table 10:** Mercury levels detected in fish samples from 17 municipalities in the Brazilian Amazon, 2021–2022.

Municipality (State)	N	No. of Species	Mean Hg μg/g (S.D.)	Median Hg	Min–Max Hg	Mean Hg μg/g Carnivorous (n)	Mean Hg μg/g Noncarnivorous (n)	% ≥0.5 μg/g
Altamira (PA)	43	13	0.30 (0.37)	0.21	0.0–1.55	0.46 (25)	0.08 (18)	14
Belém (PA)	70	24	0.20 (0.33)	0.08	0.0–2.39	0.29 (46)	0.03 (24)	8
Boa Vista (RR)	75	27	0.55 (0.65)	0.41	0.0–3.56	0.87 (43)	0.12 (32)	40
Humaitá (AM)	60	20	0.36 (0.53)	0.14	0.0–2.34	0.65 (25)	0.15 (35)	25
Itaituba (PA)	71	24	0.29 (0.39)	0.09	0.0–1.63	0.65 (26)	0.08 (45)	21
Macapá (AP)	73	25	0.17 (0.24)	0.09	0.0–1.24	0.28 (42)	0.03 (31)	11
Manaus (AM)	51	18	0.42 (0.53)	0.16	0.0–2.18	0.85 (21)	0.12 (30)	27
Maraã (AM)	48	15	0.12 (0.12)	0.08	0.0–0.52	0.33 (6)	0.10 (42)	2
Oiapoque (AP)	41	12	0.19 (0.28)	0.08	0.0–1.13	0.25 (32)	0.0 (9)	12
Oriximiná (PA)	71	21	0.20 (0.30)	0.06	0.0–1.25	0.47 (21)	0.09 (50)	14
Porto Velho (RO)	88	28	0.45 (0.82)	0.16	0.0–4.73	0.85 (40)	0.13 (48)	26
Rio Branco (AC)	78	25	0.58 (0.97)	0.15	0.0–4.64	1.06 (40)	0.08 (38)	36
Santa Isabel do Rio Negro (AM)	24	16	0.70 (0.51)	0.51	0.0–3.22	0.95 (16)	0.19 (8)	50
Santarém (PA)	70	20	0.14 (0.23)	0.03	0.0–1.13	0.35 (25)	0.02 (45)	7
São Félix do Xingú (PA)	68	22	0.50 (0.69)	0.30	0.0–3.5	0.70 (40)	0.22 (28)	29
São Gabriel da Cachoeira (AM)	32	11	0.54 (0.50)	0.43	0.0–2.25	0.67 (25)	0.05 (7)	50
Tefé (AM)	47	16	0.13 (0.15)	0.05	0.0–0.65	0.3 (15)	0.05 (32)	2

S.D.—standard deviation.

## Data Availability

Not applicable.
